# Lighting Up T Lymphocyte Signaling with Quantitative Phosphoproteomics

**DOI:** 10.3389/fimmu.2017.00938

**Published:** 2017-08-09

**Authors:** Candelas Álvarez-Salamero, Raquel Castillo-González, María N. Navarro

**Affiliations:** ^1^Departamento de Medicina, Universidad Autónoma de Madrid, Madrid, Spain; ^2^Instituto de Investigación Sanitaria del Hospital Universitario de La Princesa, Madrid, Spain; ^3^Centro de Biología Molecular Severo Ochoa, Consejo Superior de Investigaciones Científicas, Madrid, Spain

**Keywords:** T lymphocyte, signaling, phosphoproteomics, phosphorylation, mass-spectrometry

## Abstract

Phosphorylation is the most abundant post-translational modification, regulating several aspects of protein and cell function. Quantitative phosphoproteomics approaches have expanded the scope of phosphorylation analysis enabling the quantification of changes in thousands of phosphorylation sites simultaneously in two or more conditions. These approaches offer a global view of the impact of cellular perturbations such as extracellular stimuli or gene ablation in intracellular signaling networks. Such great potential also brings on a new challenge: to identify, among the thousands of phosphorylations found in global phosphoproteomics studies, the small subset of site-specific phosphorylations expected to be functionally relevant. This review focus on updating and integrating findings on T lymphocyte signaling generated using global phosphoproteomics approaches, drawing attention on the biological relevance of the obtained data.

## Introduction

**Figure 1:**
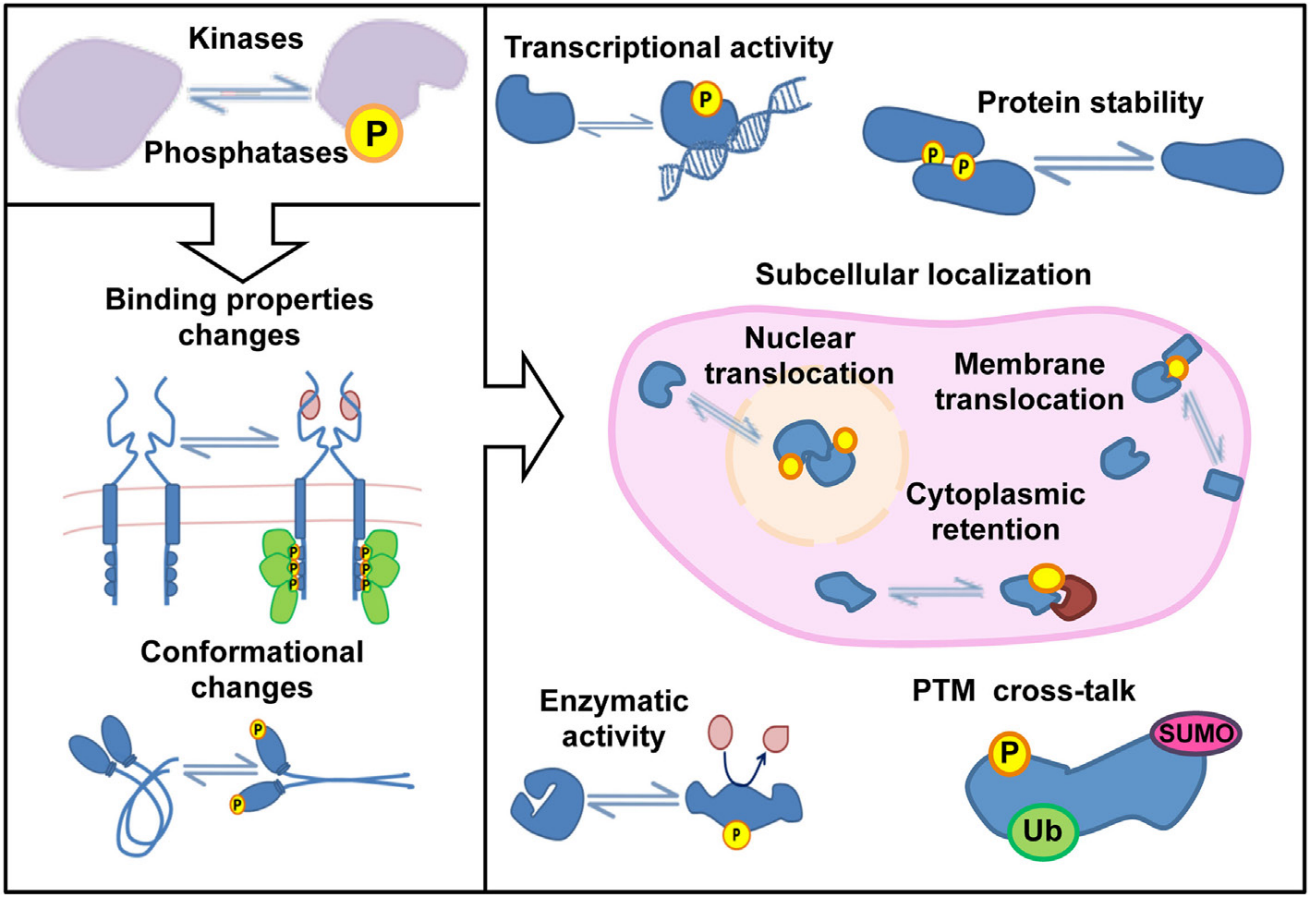
Protein functions regulated by phosphorylation.

The signaling cascades triggered in response to both extracellular and intracellular stimuli are often controlled by post-translational modifications (PTMs). Protein phosphorylation is the most abundant and best characterized PTM in eukaryotic cells. Phosphorylation involves the covalent attachment of an inorganic phosphate group to amino acid residues, and it is a reversible process determined by the balanced activity of kinases and phosphatases (1, 2). Site-specific phosphorylations (p-site) cause changes in conformation or binding properties in proteins that control essential features for their cellular function such as enzymatic activity, subcellular localization, or stability (Figure 1). Most of the phosphorylations in eukaryotic cells occur in serine, threonine, and tyrosine residues with a Ser:Thr:Tyr ratio of 81:17:2 (3, 4), and 30–50% of the cellular proteome is estimated to be phosphorylated in one or more residues at some point (5). The human genome encodes more than 500 kinases, encompassing one of the largest enzyme families, and over 100 phosphatases (6, 7). Thus, approximately 3% of the human genome is invested in the regulation of protein phosphorylation. All together, these numbers reflect the evolutionary success of phosphorylation–dephosphorylation reactions to transmit molecular information in eukaryotic cells. Phosphorylation-induced changes in protein properties propagate signaling events across phosphorylation-controlled networks, which converge and cooperate to govern fundamental processes including cell division, proliferation, migration, differentiation, and survival. Alterations in phosphorylation-mediated signaling pathways have been associated with different pathological conditions including cancer and inflammatory diseases (8, 9). Accordingly, kinases are one of the most “popular” drug targets, with more than 3,000 FDA-approved compounds or in diverse phases of clinical trials (10).

The study of site-specific phosphorylations and kinase-substrate networks has been traditionally approached using two-dimensional gels, ^32^P radioactive labeling, and antibody-based techniques such as western blotting, immunohistochemistry, ELISA, or flow cytometry. These approaches have generated invaluable knowledge on signaling cascades over the past years, but they do not offer a comprehensible, global view of the phosphoproteomic profile. More often than not, these approaches cannot identify the exact phosphorylation site. In our effort to understand intracellular communication, signaling components have been isolated and cascades have been “linearized.” However, the road from stimuli to changes in cellular functions is more complex. For example, a single stimulus often triggers simultaneously different pathways that synergize and/or antagonize in the regulation of specific cellular functions. In addition, signaling cascades often activate intrinsic positive and negative feedback mechanisms. The complexity of the phosphorylation networks is further increased by the fact that a particular protein can hold different phosphorylation sites, which may be targeted by different kinases and phosphatases. These regulatory mechanisms expand protein function, allowing a particular protein to control different activities and at the same time, to integrate different signals into coherent cellular responses. Thus, the identification and quantification of the vast amount of phosphorylation events that occur in a cell require unbiased techniques to complement traditional biochemical approaches used to study kinases and substrates on an individual basis. In this context, a recent breakthrough is the application of mass-spectrometry (MS) based quantitative phosphoproteomics to study signaling networks. This experimental approach has expanded the scope of phosphorylation analysis, enabling the simultaneous identification and quantification of changes in thousands of phosphorylation sites between two or more conditions. This powerful approach provides a global and unbiased view of the impact of natural or experimental perturbations such as gene ablation or extracellular stimuli. This great potential comes together with a new challenge: to identify, among the thousands of phosphorylations found in global phosphoproteomics studies, the small subset of site-specific phosphorylations that will be functionally relevant for the control of a specific cellular response. Starting in the last decade, an increasingly large number of studies have applied these techniques to interrogate signaling pathways in lymphocytes. Here we recapitulate how knowledge on T lymphocyte signaling has been lighted up using global phosphoproteomic approaches.

## Phosphoproteomics Workflows

**Figure 2:**
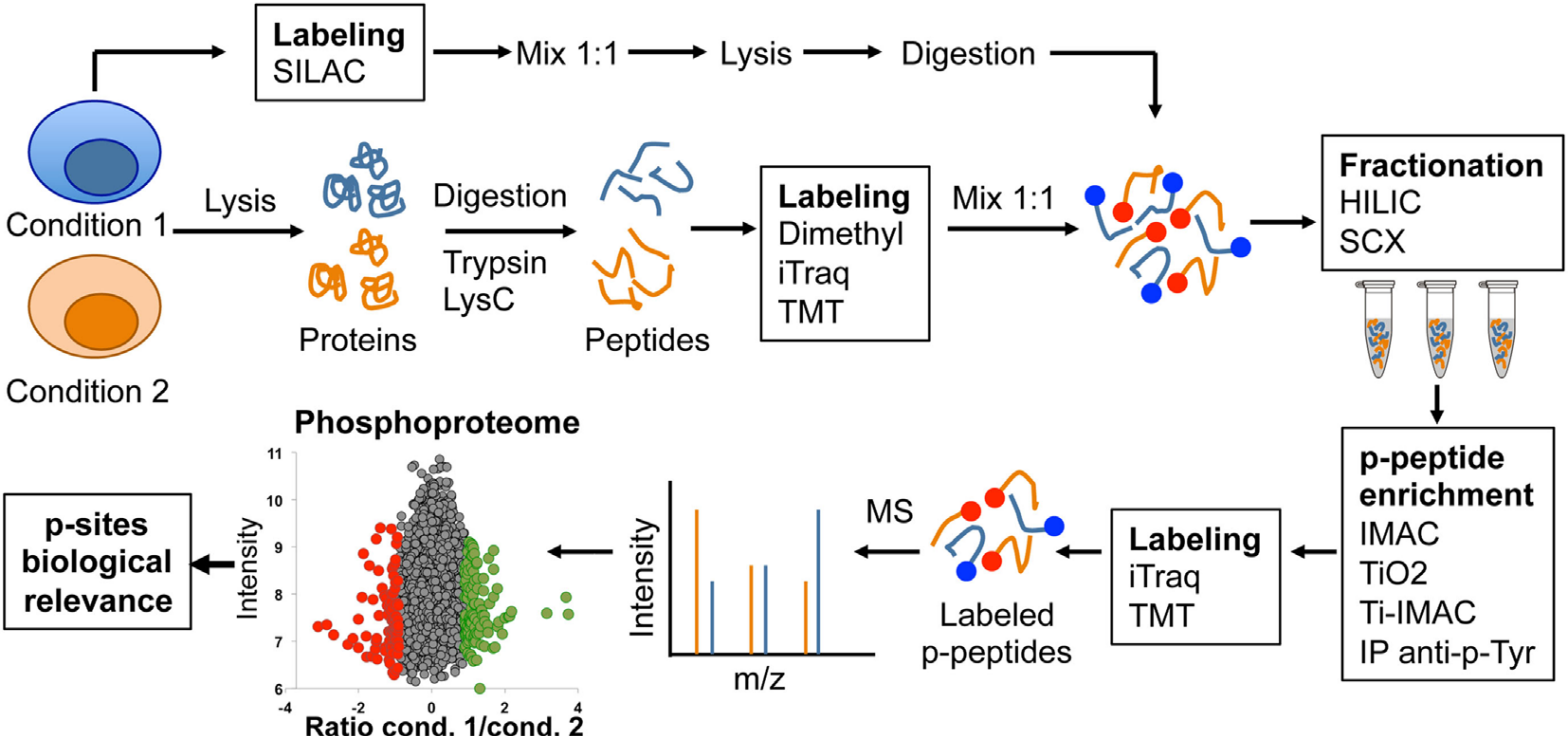
Graphic representation of typical phosphoproteomics workflow.

The continuous improvement of the technical state of the art has been crucial for the recent revolution in the application of phosphoproteomics to study lymphocyte signaling. Different techniques and protocols have been reviewed in detail elsewhere (11–13). Briefly, a typical phosphoproteomic workflow begins with protein extraction from biological samples and enzymatic digestion to generate peptides that can be detected by MS (Figure 2). The complex peptide mixtures generated are often subjected to column chromatography to generate fractions with different peptide mixtures based on their chemical properties. Sample fractionation is a key step to reduce complexity and to increase peptide identification later on in MS. Strong cation exchange and hydrophilic interaction liquid chromatography are the most common types of fractionation used in phosphoproteomic workflows. Phosphorylated peptides (p-peptides) typically represent 20% of peptides in total cell extracts. To increase the probability of MS to detect p-peptides, samples are subjected to different strategies of p-peptide enrichment. Some examples include immobilized metal-ion affinity chromatography (IMAC) or metal oxide affinity chromatography (often titanium dioxide, TiO_2_) (Figure 2). The p-peptides recovered using these enrichment matrices typically reflect the same Ser:Thr:Tyr ratio (81:17:2) reported in whole cell lysates (3, 4). Thus, p-peptide enrichment using IMAC or TiO_2_ does not increase detection of p-Tyr events. Since p-Tyr events are particularly important in T lymphocytes cascades such as the T cell receptor (TCR) signaling pathway (14, 15), immunoprecipitation using anti-phosphotyrosine antibodies at protein or peptide level have been used to enrich p-Tyr events (16). A caveat to this approach is that anti-p-Tyr antibodies recognize specific sequences and/or conformations of p-Tyr and surrounding amino acids. Thus, these antibodies do not recognize all p-Tyr events, introducing a bias to the generated data. Notably, recent studies have applied combinations of two or more p-peptide enrichment strategies to increase the coverage of cellular phosphoproteomes. In addition to the described strategies for enrichment at peptide level, phosphorylated proteins (p-proteins) can be purified using antibodies or commercially available kits prior to digestion.

Following p-peptide or p-protein enrichment, samples are subjected to analysis using reversed-phase liquid chromatography coupled to tandem MS. Due to the stochastic nature of peptide sampling by MS, different methods have been developed to achieve maximal accuracy in the quantification of changes in site-specific phosphorylations produced as a result of cell perturbations. The most commonly used methods for comparison and accurate quantification of p-peptides rely on the use of differential isotopic or isobaric labeling. Using these methods, proteins or peptides from different conditions are labeled using compounds with near identical chemical properties yet each one containing a unique isotopic composition, resulting in slightly different masses that can be distinguish by MS. Subsequently, samples from different conditions can be combined and simultaneously analyzed by MS. Stable isotope labeling strategies such as SILAC, dimethyl labeling, isobaric tagging reagents such as isobaric tags for relative and absolute quantitation (iTraq) or tandem mass tagging (TMT) labeling are often used in phosphoproteomics workflows to achieve maximal accuracy in the quantification (Figure 2). The choice of labeling method largely depends on the experimental design, cell type, and the amount of peptide to label. For actively dividing cells such as cell lines, the preferred method is SILAC labeling. This method allows the combination of up to three conditions at a very early stage during sample processing, even before cell lysis, reducing variability between conditions due to sample processing and providing maximal accuracy in p-peptide quantification (17). However, SILAC requires up to five divisions for complete protein labeling, thus not suitable for non-dividing cells, tissues, human samples, or experimental setups in which keeping cells separated under different conditions during five divisions is not desired. Differential dimethyl labeling is performed after lysis and digestion, and allows the labeling of large amounts of peptide from two to three conditions with a small cost (18). Using this strategy, conditions can be mixed before fractionation and p-peptide enrichment. Finally, iTraq and TMT methods can be used to simultaneously compare 8–10 different conditions. However, they are designed for labeling small quantities of peptide, and thus, they are commonly used after p-peptide enrichment protocols (13). Continuous improvements in MS sensitivity are increasing robustness between runs, allowing the application of label-free approaches for quantitative phosphoproteomics (16). Despite the constant improvement in workflows and MS sensitivity, a general drawback of global phosphoproteomics is that it requires a considerable amount of starting material, which is often difficult to obtain from small, low abundant cells like primary lymphocytes.

**Figure 3:**
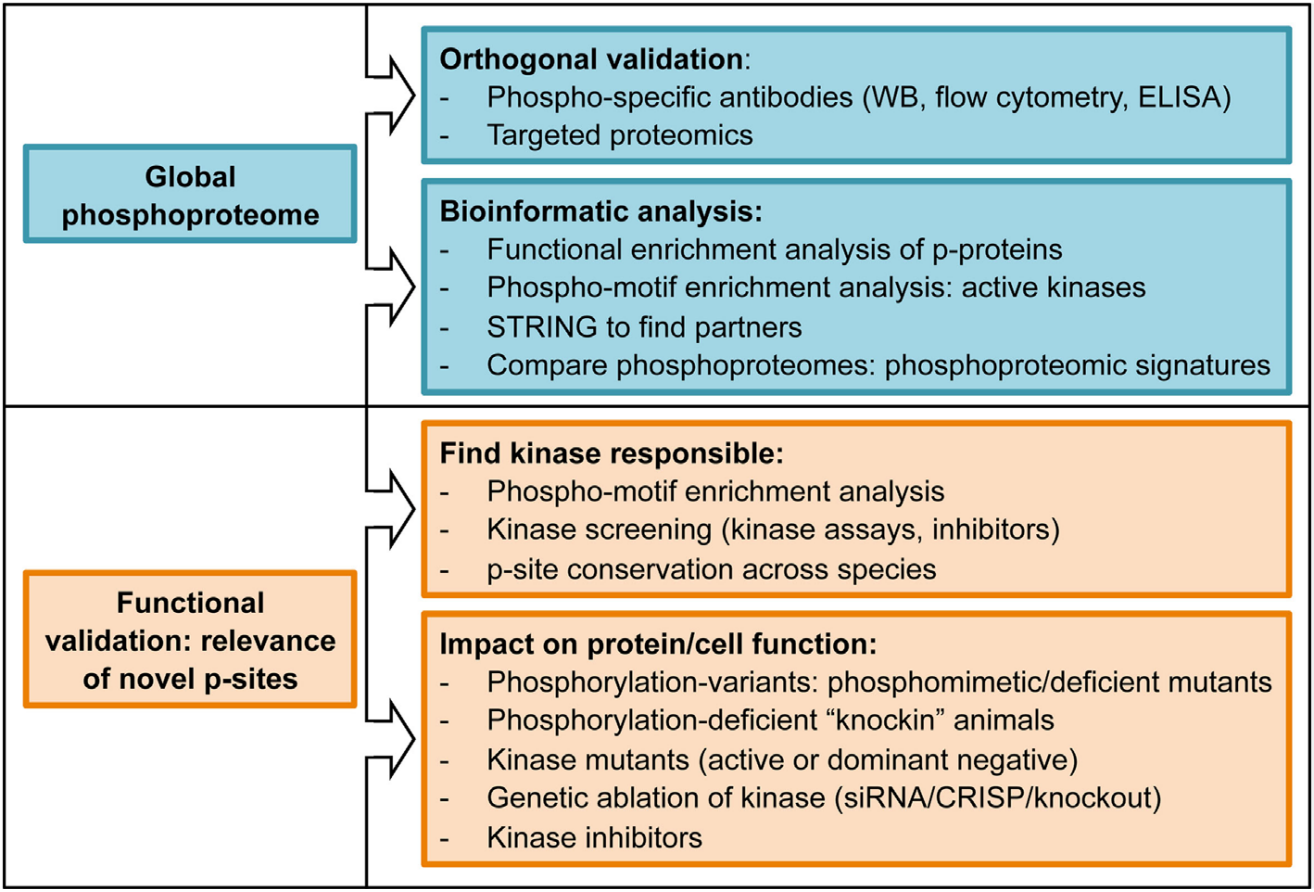
List of experimental approaches commonly used to determine biological relevance of phosphoproteomics datasets.

To summarize, phosphoproteomic workflows and MS analysis yields a global view of cellular phosphoproteomes with accurate quantification of changes in thousands of p-sites between two or more conditions. These changes require validation using alternative techniques (orthogonal validation), often antibody-based techniques such as western blotting using anti-phospho-specific antibodies. Finally, the challenge is to identify and characterize, among the whole phosphoproteome, the site-specific phosphorylations that are relevant for cellular functions. Some examples of experimental strategies followed by different authors to determine functional relevance of the data reported in phosphoproteomic studies can be found in Figure 3 and (11, 19).

## TCR Signaling by Global Phosphoproteomics

The recognition of peptide/major histocompatibility complexes (p/MHCs) by the TCR on the surface of antigen-presenting cells is a key event during the adaptive immune response. The initial events of TCR signaling are mediated by cytosolic tyrosine kinases and adaptors that couple antigen recognition to a network of serine–threonine kinases that propagate the signal from the cell membrane to the nucleus, promoting the transcriptional changes that support T cell differentiation and acquisition of effector functions (20). Several studies have approached TCR signaling using phosphoproteomic approaches in the human T cell line Jurkat, which is a major source of knowledge regarding TCR signaling. Pioneering studies were developed as proof-of-concept experiments to examine whether global phosphoproteomics could be used to interrogate TCR signaling (4, 21, 22). Key phosphorylation events on tyrosine residues in CD3 chains, Lck or ZAP70 were observed in anti-CD3-stimulated Jurkat cells (21, 22). In addition, well-characterized phosphorylations regulated by TCR stimulation on NFAT, Erk, and Stathmin were detected in primary cytotoxic T cells (CTLs) after cognate-peptide stimulation (4) and in anti-CD3-stimulated human peripheral blood lymphocytes (hPBLs) (23). These studies verified the detection of canonical TCR-dependent signaling events, and validated the use of global phosphoproteomic approaches to generate new knowledge on TCR signaling pathways. Table 1 summarizes key information (cell type, stimulation, number of p-sites found) of the different global phosphoproteomic studies that have explored TCR signaling.

**Table 1:** Global phosphoproteomic studies exploring T cell receptor (TCR) signaling.

Focus	Enrichment	Labeling	Cell model	TCR stimulation	Reference	Total p-sites
TCR, ZAP70	IP p-Tyr	SILAC; label free	Jurkat, ZAP70 null	CD3/CD4; 0–10 min	(21)	168
TCR	IP p-Tyr	iTraq	Naïve CD4, B6g7 vs NOD mice strains	CD3; 5 min	(24)	77
TCR, SLP76	IP p-Tyr	SILAC	Jurkat, SLP76 null	CD3; 0–20 min	(25)	758 p-proteins
TCR	IP p-Tyr	SILAC	Jurkat	CD3/aCD28; 0–1 min	(26)	700
TCR	p-S/T/Y (IMAC)	SILAC	Jurkat	CD3; 15–60 min	(22)	10,665
TCR	p-S/T/Y (IMAC)	SILAC	mCTLs	Cognate peptide; 60 min	(4)	2,081
TCR	p-S/T/Y (TiO_2_)	iTraq	hPBLs	CD3; 5 min	(23)	2,814
ZAP70	IP p-Tyr	SILAC	Jurkat- ZAP70^AS^ mutant (PP1 inhibitor sensitive)	CD3/CD4; 0–10 min; ±PP1 treatment	(27)	905
LAT	p-S/T/Y (TiO_2_)	SILAC	Jurkat, LAT null	CD3; 0–20 min	(28)	11,454
SLP76	IP p-Tyr	SILAC	Jurkat, SLP76 null	CD3/CD4; 0–10 min	(29)	270
SLP76	IP p-Tyr	SILAC	Jurkat, SLP76 mutant	CD3/CD4; 0–10 min	(30)	934
PKD2	p-S/T/Y (IMAC + TiO_2_)	SILAC	mCTLs, PKD2 knockout	Cognate peptide; 5 min	(31)	15,871
PKD2	p-protein	Label free	Thymocytes, PKD2/PKD3 double knockout	CD3; 2 min	(32)	na
Vav1	IP p-Tyr	Label free	Jurkat, Vav1 null	CD3/CD4; 0–10 min	(33)	652
Erk	IP p-Tyr	SILAC	Jurkat, MEK inhibitor	Inhibitor pre-treatment, CD3/CD4; 0–10 min	(34)	322

*na, not applicable; h, human; m, mouse; iTraq, isobaric tags for relative and absolute quantitation; NOD, non-obese diabetes; IMAC, immobilized metal-ion affinity chromatography; CLTs, cytotoxic T cells; PKD, protein kinase D*.

### Focus on Tyrosine Phosphorylation

**Figure 4:**
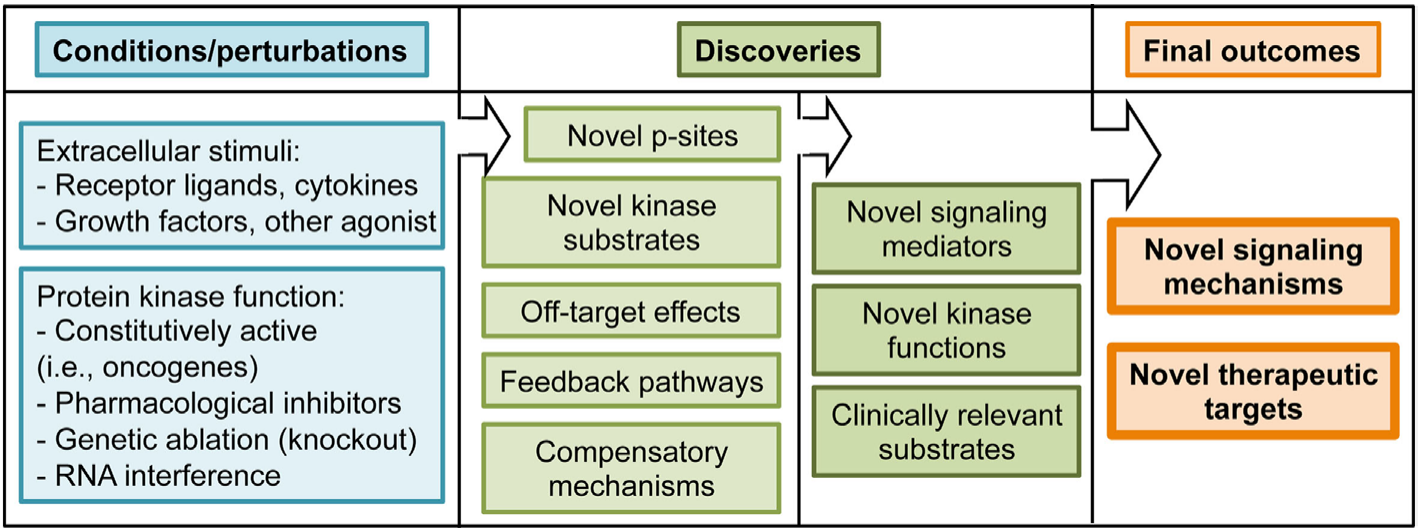
Summary of experimental conditions and potential outcomes of phosphoproteomic studies.

The TCR signaling cascade sets off phosphorylation events on tyrosine residues that have been characterized during the past 20 years (14). Using p-Tyr enrichment protocols, a pioneering study in anti-CD3 stimulated Jurkat cells identified a positive feedback loop of ZAP70 on Lck activity (21). This work constitutes an example of how feedback mechanisms emerge when signaling cascades are interrogated in a global manner (Figure 4). The positive feedback pathways on early TCR signaling identified through phosphoproteomics opened new lines of research that have been further examined in later studies (27, 34). In addition to feedback pathways, global phosphoproteomics have the potential to identify novel players downstream TCR triggering (Figure 4). For example, a p-Tyr enrichment study focused on early waves of tyrosine phosphorylation in Jurkat cells. This work identified 758 phosphorylated proteins, of which 141 were responsive to anti-CD3 stimulation (25). It also revealed that the protein Themis was phosphorylated on Tyr residues shortly after TCR triggering in a Lck- and LAT-dependent manner, and that its expression was required for interleukin 2 (IL-2) production (25). Themis had been recently identified as a key player during thymic T cell development (35, 36). Subsequent work using Themis deficient mice and phosphorylation-deficient Themis mutants has firmly established that Themis phosphorylation, and its association with the adaptor protein Grb2 and the phosphatase SHP1, are key components of the early TCR activation that dictates the signaling threshold during positive and negative selection in the thymus (37, 38). These studies illustrate how unbiased phosphoproteomics can unveil phosphorylation events that turn out to be crucial signaling events upon additional characterization. In addition to Themis, several proteins involved in mRNA regulation were found phosphorylated on tyrosine residues within the first 30 s after TCR stimulation (25). This finding relocates transcription and translation, often perceived as closing events of signaling cascades, to the top of the TCR signaling cascade. The TCR-regulated p-Tyr events have been further explored in Jurkat cells to characterize tyrosine phosphorylation in response to short (less than a minute) anti-CD3/CD28 stimulation (26). This work put together phosphorylation of activating p-sites and dephosphorylation of inhibitory p-sites as key events in the initiation of the TCR signaling pathway, showing that TCR-induced phosphorylations regulate phosphatase activity. Furthermore, this work showed a novel application of phosphoproteomics datasets, which is the implementation of mathematical modeling of signaling networks. By combining model simulation and experimental confirmation of model predictions, the TCR-mediated phosphorylation of the phosphatase PTPN6 emerged as a critical event for an early signaling boost required to launch rapid T cell responses.

As mentioned above, p-Tyr enrichment approaches require large amounts of starting material difficult to obtain from primary T cells. However, an ambitious study compared tyrosine phosphorylation after TCR signaling in *ex vivo* isolated T cells from two different mouse strains, the B6g7 and the non-obese diabetes (NOD) mice, as a strategy to find molecules responsible for hyperreactive T cells in NOD mice (24). This study revealed that TCR signaling mediators such as ZAP70, DOCK2, and SLAP exhibited lower phosphorylation levels in NOD compared with B6g7 T cells, while three different p-Tyr residues in the kinase TXK displayed increased phosphorylation in NOD T cells in response to TCR stimulation. Notably, the precise quantification obtained using MS techniques revealed small differences, which were undetectable by conventional biochemical analyses. Even though further experiments will be required to determine if these changes are involved in the development of the diabetes in NOD mice, this study pioneered the application of phosphoproteomic screenings to compare genetically modified primary T cells.

### Global p-Ser/Thr/Tyr Approaches

Unbiased phosphopeptide enrichment protocols have been used to obtain a comprehensive view of the global impact of TCR stimulation on phosphorylation networks (Figure 2). A ground-breaking article in 2009 applied a large-scale, unbiased, quantitative phosphoproteomic approach to anti-CD3-stimulated Jurkat cells to uncover novel members of the TCR signaling cascade that participate in the T cell activation process (22). This study identified more than 10,000 p-sites on 3,000 proteins, 696 of which were regulated by TCR stimulation including 60 previously unknown TCR-regulated p-sites. This work showed that phosphorylated proteins in T cells have diverse biological functions, including transcriptional regulation, actin dynamics regulation, endocytosis, or mRNA splicing. This work took advantage of another powerful application of phosphoproteomic approaches, which is the identification of kinase substrates using small molecule inhibitors of kinase activity (Figure 4). The treatment of anti-CD3-stimulated Jurkat cells with a MAPK inhibitor identified 143 known Erk substrates, together with 54 putative Erk substrates such as the B cell lymphoma 11B (BCL11B). More importantly, MAPK-mediated phosphorylation of BCL11B and subsequent sumoylation and ubiquitination was required for gene expression during positive selection in the thymus (39, 40). As T cells are a powerful model of proliferative and metabolically active cells with features common to tumor cells, it is not surprising that several p-sites first reported in Ref. (22) bearing roles in cell cycle progression and proliferation have been functionally validated in the cancer research field (41, 42). Overall, site-specific phosphorylation events reported in Mayya et al. study have been explored in more than 20 publications, validating the potential of phosphoproteomic approaches to generate novel, hypothesis-driven experimental work.

The development and optimization of novel protocols, together with the increased sensitivity of MS has allowed the application of global phosphoproteomic approaches to the study of TCR signaling in primary CTLs obtained from TCR transgenic mouse. Of note, the use of TCR transgenic T cells enabled a more physiological manner of TCR triggering using cognate p/MHC complexes (4). In this study, we reported more than 2,000 site-specific phosphorylations on CTLs, with more than 400 up- or down-regulated in response to TCR stimulation. Bioinformatic analysis of enriched protein functions in the CTL phosphoproteome revealed that most of the proteins phosphorylated in steady-state are involved in transcriptional regulation, RNA post-transcriptional modifications, and other nucleic acid binding processes, supporting the idea that the main goal of constitutive signaling networks is to maintain specific transcriptional programs that allow T cell function. This work showed that not only TCR-regulated phosphorylations exert a prominent role in T cells. For example, using phosphorylation-deficient mutants, we showed that intrinsic phosphorylation and nuclear exclusion of the class II histone deacetylase 7 was a crucial event to maintain the transcriptional profile required for CTL proliferation, IL-2 response, and IFNγ secretion. This work took advantage of another valuable application of the global phosphoproteomic approaches, which is the possibility of performing motif enrichment analysis to predict the pool of active kinases in a given cell (Figure 3). This knowledge can be used to predict the impact of small molecule inhibitors of kinase activity on intracellular signaling networks, or to determine if a kinase of interest is present and active in target cells. These predictions could be relevant to bring small molecule inhibitors from the bench to the bedside (Figure 4). Motif enrichment tools were also used to analyze the phosphoproteome of hPBLs in response to anti-CD3 stimulation. Of the 2,814 p-sites reported in PBLs, 517 were regulated by TCR stimulation, and defined CaMK, Rsk, protein kinase D (PKD), and Erk1/2 as the core TCR-regulated kinases (23).

Global phosphoproteomics has also been used to uncover signaling hubs controlled by known TCR signaling components using Jurkat cells deficient for the expression of the protein of interest. Moreover, this approach was taken further in studies that use primary T lymphocytes obtained from knockout animals (Table 1). The role of the tyrosine kinase ZAP70 was one of the earliest events in the TCR signaling cascade examined by phosphoproteomics (21, 27). These studies showed that ZAP70 deficiency in Jurkat cells affected not only downstream phosphorylations, but also upstream phosphorylations such as the tyrosine residues on CD3 ITAM motifs, indicating the existence of ZAP70-dependent negative feedback pathways (21). These feedback mechanisms were further explored using ZAP70-deficient Jurkat cells reconstituted with a ZAP70 mutant form (ZAP70^AS^), which is susceptible to inhibition using an analog of the kinase inhibitor 4-amino-1-*tert*-butyl-3-(*p*-methylphenyl)pyrazolo[3,4-d]pyrimidine (27). These experimental setting allowed the study of acute deletion of ZAP70 kinase activity on TCR signaling. These studies confirmed that ZAP70 promotes negative feedback mechanisms targeting Lck and ZAP70 itself, revealing that negative pathways emerge from very early signaling events downstream of the TCR. Evidences of negative feedback pathways were also found in a phosphoproteomic study focused on one of the best known ZAP70 substrates, LAT, portraying LAT as a negative regulator of upstream signaling events (28). These results may resolve the apparent paradox of autoimmune pathology brought upon by defects on LAT, which is an essential component in TCR signaling (43).

The signaling hub controlled by another ZAP70 substrate, SLP76, has been extensively interrogated using global phosphoproteomic approaches. The studies performed in SLP76-deficient Jurkat cells showed, as expected, that a large cohort of the TCR signaling events depended on the LAT/SLP76 node. However, these studies also showed evidences of SLP76-independent events (25, 29, 30). Going further on the TCR signaling cascade, phosphoproteomics studies in Vav1-deficient Jurkat cells revealed a novel, Vav1-dependent negative feedback pathway. Indeed, Vav1 deletion increased phosphorylation of several TCR proximal kinases such as Lck and ZAP70. Subsequently, phosphorylation of CD3 ITAMs, Itk, or Erk was also increased in absence of Vav1 (33). The existence of several negative feedback pathways illustrates the intense pressure on early TCR signaling events that is required to distinguish between self- and foreign antigens. However, T cells also need mechanisms to ensure rapid responses to foreign antigens. In this context, feedback pathways such as the positive regulation of Erk on Lck activity have been explored using phosphoproteomics. TCR signaling was evaluated in Jurkat cells pre-treated with a MEK1/2 inhibitor that prevents Erk1/2 activation. In addition to decreased Lck and CD3 phosphorylation, this study extended the magnitude of Erk1/2 feedback pathway, showing that Erk1/2 activity was required for the phosphorylation and activation of proteins involved in actin dynamics such as Vav1 and WASP, and proteins involved in integrin signaling such as focal adhesion kinase (34).

The TCR signaling cascade relies on the generation of lipid mediators such as diacylglycerol (DAG) to transmit the signal originated at the cell surface to the cytosol. Local changes in the concentration of DAG are sensed by serine/threonine kinases of the protein kinase C (PKCs) and the PKDs families (20). The signaling network controlled by PKD in T cells was interrogated using global phosphoproteomics in PKD2-deficient primary CTLs (31). In this work, we compared CTLs obtained from wild-type and PKD2-knockout animals. As a strategy to find PKD2 substrates, the PKD consensus motif was searched among the down-regulated phosphorylations found in PKD2-knockout animals, producing a list of 73 PKD substrate candidates to mediate T cell activation and cytokine secretion in T cells (44, 45). Searching for PKD substrates, a different experimental approach was recently developed using thymocytes from PKD2/PKD3 double knockout mice. In this study, phosphorylated proteins were enriched followed by 2D-DIGE and protein spots analyzed by MS. This study identified the phosphatase SHP1 (*Ptpn6*) Ser557 residue as a PKD substrate. Remarkably, this study reported the generation of a “knockin” mouse bearing a phosphorylation-deficient SHP1 variant (S557A mutation), to demonstrate that SHP1-Ser557 phosphorylation is required for optimal T cell differentiation in the thymus (32).

An interesting yet unsolved question in the T lymphocyte signaling field is whether effector T cells use unique signaling components that contribute to the distinct effector T cell response signaling. Quantitative phosphoproteomics is a powerful tool to provide insights into this question. For example, while effector T cell immune synapses (IS) recruit in CD28 and PKCθ, regulatory T cells (Tregs) IS are enriched in CTLA4 instead of CD28, lacking PKCθ (46). The conspicuous absence of PKCθ argues that the TCR signaling cascade must be different in Tregs. CTLA4, a key molecule in Treg-mediated immune suppression, was shown to interact with the kinase PKC-η. Moreover, PKC-η-deficient Tregs displayed defective suppressive activity (47). To get insights into the molecular mechanisms regulating CTLA4/PKC-η-mediated Treg suppression, quantitative phosphoproteomics was used to compare anti-CD3 stimulated wild-type and PKC-η deficient induced Tregs. In this study, phosphorylation of the kinase PAK2 and the G protein-coupled receptor kinase-interacting protein 2 (GIT2) were reduced in PKC-η deficient Tregs. PAK2 and GIT2 are isoforms of proteins heavily involved in focal adhesion disassembly, cellular motility, and synapse formation in neurons (48, 49). In Tregs, these molecules were found associated to CTLA4 and PKC-η. Moreover, impaired phosphorylation and activation of PAK2 in PKC-η null Tregs was associated with reduced ability to serially engage APCs, providing a potential mechanistic basis for the decreased suppressive function of these cells in the absence of PKC-η. This pathway represents a potential therapeutic target for inhibiting Treg-mediated suppression in tumors. These latest works are stepping stones toward the validation of phosphoproteomics approaches to address physiologically relevant questions. To summarize, the application of global phosphoproteomics approaches has identified novel components of TCR signaling, and evidenced that feedback pathways and intrinsic phosphorylation networks are critical for T cell function. In addition, these studies have identified hundreds of novel TCR-regulated p-sites that now can be subjected to targeted, hypothesis-based approaches for further functional validation (Figure 4). Site-specific phosphorylations reported in phosphoproteomics studies represent an opportunity for therapeutic intervention, since follow up research will generate tools to boost T cell activity against tumors or to dampen T cell function in autoimmune and inflammatory conditions.

## Other Signaling Processes in T Lymphocytes

### IL-2 Signaling Pathway

T cell activation depends on TCR signaling pathways triggered by antigen recognition. In addition, T cell differentiation and the acquisition of effector functions are strongly influenced by the cytokine milieu generated during the inflammatory response. Secreted mediators such as cytokines, chemokines, and growth factors are critical intercellular messengers that mediate the generation of effector T cells. In this context, IL-2 is the main controller of CD4 and CD8 clonal expansion and differentiation (50). In addition to its critical role as T cell mitogen, the study of the IL-2-dependent signaling network is very relevant to understand a host of human malignancies. For example, the administration of high doses of recombinant IL-2 is currently being used to boost T cell activity against metastatic melanoma and other tumors (51), while low doses of IL-2 are being used to increase Treg function in autoimmune and inflammatory conditions (52). Individual aspects of the IL-2 signaling pathway such as the activation of Jak/STATs, Ras/MAPK, and PI3K/Akt have been extensively interrogated using conventional biochemistry. Recently, a global view of the IL-2 signaling pathway is emerging from data obtained using global phosphoproteomic approaches. IL-2-regulated phosphorylations on tyrosine residues were analyzed in the human T cell line Kit225, identifying protein, and lipid phosphatases (SHP2, SHIP1, and SHIP2, respectively) as targets of the IL-2 signaling cascade (53). Further analysis of the IL-2-regulated nuclear phosphoproteome revealed that the ATP-citrate liase ACLY, which is an acetyl-CoA generating enzyme with a relevant role in tumor cell proliferation, was phosphorylated on Ser455 in response to IL-2 stimulation in an Akt-dependent manner. Moreover, the overexpression of phosphomimetic mutants of ACLY-Ser455 increased proliferation of an epithelial cell line. This work identified ACLY as a novel IL-2-regulated protein whose phosphorylation promotes cell proliferation (54).

The IL-2 signaling pathway has been also examined in primary CTLs. Upon stimulation of murine CTLs with IL-2 for 15 min, phosphorylation was induced in more than 700 p-sites and reduced on 220 p-sites, revealing the mRNA binding protein YBX1-Ser100 as a novel component of the IL-2 signaling pathway (55). In this exhaustive study, the role of Jak1/3 and Src-kinases in CTL signaling pathways was examined using specific inhibitors of kinase activity. This comparative analysis identified previously unknown Jak-independent, Src-regulated mechanisms downstream the binding of IL-2 to its surface receptor. These experiments offered a global view of the impact of kinase inhibitors on CTL phosphoproteome, showing that Src-kinases Lck and Fyn are active in CTLs but not regulated by the IL-2/Jak axis. More importantly, the data showed that both Src- and Jak kinases regulate mTORC1 function in CTLs and surprisingly, Src-kinases rather than IL-2/Jak are the main controllers of Akt function in CTLs (55). Thus, these experiments identified novel signaling mechanisms that can be exploited to modify both T cell effector function as well as their fate. Phosphoproteomics have also been used to elucidate how two mitogens, IL-2 and IL-15, exert unique roles on T cells despite signaling through the same receptors (IL-2Rγc and IL-2Rβ). The quantitative comparison of IL-2- and IL-15-regulated phosphoproteomes showed that early signaling events mostly overlap (56, 57). Thus, the data suggest that IL-2 and IL-15 signaling pathways are quantitatively but not qualitatively different, and that the unique biological activity that each cytokine exerts is due to the sustained signal maintained by IL-2 but not by IL-15 (58). Table 2 collects key information on the different global phosphoproteomic studies exploring cytokine signaling pathways in T lymphocytes.

**Table 2:** Global phosphoproteomic studies exploring cytokine and chemokine signaling pathways, and T lymphocyte-mediated disease.

Focus	Enrichment	Labeling	Cell model	Stimulation	Reference	Total p-sites
IL-2	IP p-Tyr	SILAC	Kit225	IL-2; 5 min	(53)	1,392 p-proteins
IL-2; IL-15	IP p-Tyr	iTraq	F15R-Kit	IL-2 or IL-15; 15 min	(56)	85
IL-2, IL-15	IP p-Tyr	SILAC	Kit225	IL-2 or IL-15; 5 min	(57)	1,255 p-proteins
IL-2, IL-15	p-S/T/Y (TiO_2_)	SILAC	Kit225	IL-2 or IL-15; 5 min	(59)	85
IL-2	p-S/T/Y (TiO_2_)	SILAC	Kit225, nucleus	IL-2; 5 min	(54)	8,521
IL-2	p-S/T/Y (Ti-IMAC)	SILAC	mCTLs	IL-2; 15 min	(55)	6,458
IL-2/JAKs	p-S/T/Y (TiO_2_)	SILAC	mCTLs	Jak inhibitor, 30 min; 4 h	(55)	8,839; 11,820
IL-2, Src-kinases	p-S/T/Y (TiO_2_)	SILAC	mCTLs	Src-inhibitor; 4 h	(55)	15,353
SDF1	p-S/T/Y (IMAC)	SILAC	CEM	SDF1; 5 min	(60)	4,074
HIV entry	p-S/T/Y (IMAC)	SILAC	CEM	HIV; 5 min	(61)	1,757
HIV spread	p-S/T/Y (TiO_2_)	SILAC	Jurkat	HIV-infected cells + uninfected cells (0–40 min)	(62)	28,853
ALK	p-S/T/Y (TiO_2_) + IP p-Tyr	Label free	ALCL lymphoma cell lines	ALK inhibitor; 6 h	(63)	671 p-proteins
Hematological cancer	p-S/T/Y (IMAC)	Label free	Acute myeloid leukemia, P31/Fuj; CTS; MV4-11. Lymphoma, RL; SU-DHL-6; Dohh-2. Multiple myeloma, RPMI-8226; OMP2; U266B1	na	(64)	2,434

*na, not applicable; m, mouse; IKL-2, interleukin 2; IMAC, immobilized metal-ion affinity chromatography; CLTs, cytotoxic T cells; HIV, human immunodeficiency virus; ALK, anaplastic lymphoma kinase; ALCL, anaplastic large cell lymphoma*.

### Chemokine Signaling and Human Immunodeficiency Virus (HIV) Infection

The binding of the chemokine CXCL12 (SDF1) to its receptor CXCR4 has been implicated in HIV-1 infection and cancer metastases. This signaling pathway was explored in a phosphoproteomic study using the T lymphoblast cell line CEM. This study confirmed previously known SDF1-regulated p-sites in core T cell kinases such as Akt and Erk2, and also revealed novel SDF1-responsive p-sites in proteins such as Stathmin (60). Comparison with a previous TCR-regulated phosphoproteome (22) found an interesting overlap between CXCR4 and TCR signaling pathways. Additional data on CXCR4 triggering was provided by a later study that used phosphoproteomics to study signaling cascades during HIV entry in CD4 cells (61). Phosphoproteomic analysis in CEM cells 1 min after infection revealed HIV-responsive phosphorylations that were further validated using HIV-infected human resting CD4 lymphocytes. As an example, the core T cell kinase p38 was found phosphorylated after HIV infection. More importantly, this work revealed that a regulator of cellular splicing, SRRM2, was a component of the cellular machinery that is used by the HIV for the correct splicing of *Nef* and *Tat* viral transcripts. As a consequence, SRRM2 depletion causes a change in the ratio of spliced forms, decreasing virion release. This is an example of how studying a process such as virus entry using global phosphoproteomics has unexpectedly lead to the identification of cellular proteins involved in the splicing of viral transcripts. Moreover, the authors found other 239 HIV-responsive phosphorylations on 175 different proteins, each of them with a potential role in HIV infection that merits additional investigation. The analysis of virus entry using global phosphoproteomics has been also applied to lytic gammaherpesvirus (65) and influenza virus (66).

In addition to analysis of virus entry, phosphoproteomics has been recently used to study HIV cell–cell spread (62). Changes in phosphorylation sites were studied at different time points after combining HIV-infected Jurkat cells with uninfected cells, identifying a total of 28,853 p-peptides on 5,649 proteins. This is the largest single dataset reported to date from a lymphocyte phosphoproteomic analysis. The experimental settings used in this work allowed the discrimination of site-specific phosphorylations occurring in the HIV-donor cells from those occurring in the host cells. Surprisingly, bioinformatics pathway analysis showed that TCR signaling in the donor T cells was significantly modified during HIV-1 spread. To demonstrate the relationship between TCR signaling and HIV spread, this study examined HIV infection and cell–cell spread in Jurkat variants deficient for TCR/CD3, Lck, and ZAP70. These Jurkat variants supported HIV infection and viral replication. However, when the TCR signaling-deficient infected cells were incubated with wild-type target T cells, a significant impairment in virus cell–cell spread was observed. Thus, this work has unveiled an unexpected link between TCR signaling in HIV-infected T cells and virus transmission to neighboring cells, opening the door for novel therapeutic strategies based on interference with TCR signaling to prevent HIV-1 spread. To summarize, since virus highjack the machinery of the host cell, any cellular protein required for viral entry, replication or diffusion is a potential therapeutic target. Thus, the systematic application of global phosphoproteomics to the study of viral infection can lead to the development of novel strategies to interfere with infectious processes. Table 2 collects key information on the different global phosphoproteomic studies exploring chemokine signaling pathways in T lymphocytes.

### T Lymphocyte-Mediated Diseases

Phosphoproteomics is being used to understand the role of phosphorylation networks on T cell activation, but it can also offer insights into the alterations of regulatory mechanisms that underlie the transformation processes causing lymphomas, leukemia, and other lymphocyte-mediated diseases such as inflammatory and autoimmune conditions. Although this approach has been exploited so far to study mostly B cell tumors (67), some studies have focus on T cells. For example, the phosphoproteomic signature of different hematological cancers, including T cell lymphomas, was analyzed. Principal component analysis and unsupervised hierarchical clustering of phosphoproteomic signatures grouped together cell lines from the same origin (64). The phosphoproteomic profiling of tumors can be used for diagnosis of hematological malignancies, to reach or confirm a diagnosis, but also proves the existence of conserved phosphorylation networks and signaling pathways in different types of T cell malignancies. This study also tested an interesting hypothesis, which is whether phosphorylation levels of a specific set of p-sites can be used to predict sensitivity or resistance to treatment. Using kinase inhibitors for different pathways (PI3K/mTOR, MEK, Jak), the authors determined cell viability as a proxy of sensitivity to inhibitor, and correlated this data with groups of specific p-sites. Unexpectedly, their findings showed that known kinase substrates do not correlate particularly well with sensitivity. Interestingly, they found that resistance to PI3K/mTOR inhibition correlated with a fair number of PKC sites, leading to the conclusion that acute myeloid leukemia-derived cell lines use PKC pathways to proliferate, thus explaining the resistance to PI3K/mTOR inhibition. In a similar manner, resistance to Jak inhibitors correlated with increased S6K activity, suggesting that Jak inhibitor-resistant leukemia cell lines use the mTORC1 pathway to proliferate. This study illustrates the potential of defining the phosphoproteomic signature of tumor cells to predict their resistance to anti-tumor treatments.

Phosphoproteomics has also been used to decipher molecular mechanism underlying T cell transformation as a strategy to identify potential treatments. Anaplastic large cell lymphoma (ALCL) is often driven by a constitutively active tyrosine kinase, the nucleophosmin-anaplastic lymphoma kinase (ALK). To evaluate the therapeutic potential of inhibiting ALK to treat ALCL, the effect of an ALK inhibitor was analyzed in ALCL-derived cells using phosphoproteomics (63). Among the inhibitor-responsive phosphorylations, p-peptides of an isoenzyme of the glycolytic enzyme pyruvate kinase (PKM2) were found down-regulated by ALK-inhibitor treatment. In addition, increased p-PKM2 was found in four different ALCL cell lines. *In vitro* kinase assays demonstrated that PKM2 is a substrate of ALK. Moreover, the combination of pharmacological inhibition and phosphorylation mutants of PKM2 showed that ALK-mediated phosphorylation of PKM2-Tyr105 inhibits PKM2 activity, thus promoting glycolysis, Warburg metabolism, and tumor progression. Thus, the blockage of PKM2 phosphorylation represents a potential mechanism to control cell proliferation in ALK-mediated lymphomas. Interestingly, increased PKM2 phosphorylation on Ser37 residue, which induces the expression of genes that promote the Warburg metabolism, was detected in a phosphoproteomic study in response to IL-23 stimulation in the Kit225 human T cell line (68). Hence, PKM2 phosphorylation on different residues revealed the ability of different signaling cascades to control T cell metabolism.

In addition to leukemia and lymphomas, aberrant T lymphocyte function underlies pathological conditions such as multiple sclerosis. Phosphoproteomic analysis of multiple sclerosis brain lesions detected sphingosine 1-phosphate receptor (S1P1) phosphorylation on Ser351 (69). S1P orchestrates lymphocyte migration from lymphoid organs into circulation. In the context of the disease, treatment with the S1P1 functional antagonist FTY-720 (which blocks lymphocyte trafficking) prevents multiple sclerosis relapses. The role of S1P1-Ser351 phosphorylation, a key residue for S1P1 internalization, was subsequently examined in mice expressing a phosphorylation-deficient receptor (S1P1-S5A). These mice developed severe experimental autoimmune encephalomyelitis due to autoimmunity mediated by IL 17-producing helper T cells (Th17 cells). Impaired S1P1 phosphorylation and internalization enhanced Th17 polarization, exacerbating inflammation of the central nervous system. This work illustrates the ability of global phosphoproteomics to unmask novel pathogenic mechanisms in T cell-mediated diseases such as multiple sclerosis (69). Table 2 collects key information on the different global phosphoproteomic studies exploring T lymphocyte-mediated diseases.

## PTMs-OMICS

In addition to phosphorylation, other PTMs also participate in signaling processes to control protein and cell function. The cellular scenario becomes even more complicated because the different PTMs crosstalk. As an example, phosphorylation can control the ability of proteins to undergo additional PTMs of different types (70). The advantages of global, unbiased analysis of phosphorylation networks by MS exposed in this review can be extended to other PTMs. In general, the analysis of PTMs other than phosphorylation faces the same basic challenges, including small subsets of modified proteins together with an excess of unmodified peptide compared to modified peptide. Therefore, as global phosphorylation analysis requires p-peptide enrichment, other PTMs require different types of enrichment to increase their detection by MS. This section covers some of the most recent developments on PTMs-omics that can be used to expand the knowledge on T lymphocyte signaling.

*Ubiquitination* consist in the addition of ubiquitin to a Lys residue of substrate protein. The enrichment strategy for “ubiquitome” profiling more often used is based on immunoprecipitation using an antibody that recognizes the glycine–glycine bound to the modified Lys-residue (K-ε-GG), generated after trypsin digestion of ubiquitinated proteins. Ubiquitinated sites are subsequently identified by MS (71, 72). Recently, a novel method has been developed to specifically detect substrates modified with linear polyubiquitination (polyUb): the use of internally tagged ubiquitin chain that enables subsequent enrichment by pull down and MS identification. Combined with SILAC labeling for quantification, this approach identified 1,695 potentially polyUb, and identified TRAF6 as a linear polyUb substrate of the LUBAC complex in response to TNFα treatment in HEK293T cells (73). Of note, mutations in the ubiquitination system are the underlying cause of several inflammatory diseases (9). Thus, the ubiquitome profiling of T lymphocytes will surely generate novel therapeutic targets in inflammatory conditions.

*SUMOylation* links the small ubiquitin-like modifier protein (SUMO) to target proteins at the ε-amino group of Lys residues, regulating protein function, and activity. His-tagged versions of SUMO have been expressed in HEK293T cells, allowing the enrichment of SUMOylated proteins using affinity chromatography. Then, samples are digested and immunoprecipitated with monoclonal antibody that specifically recognize SUMO remnants left after tryptic digestion of SUMOylated proteins. This experimental approach has led to the identification of 954 SUMO3-modified Lys residues, 86% of the sites previously unknown (74). A different approach employed a mutant version of SUMO, in which the residue preceding the c-terminal Gly–Gly is replaced with a Lys (SUMO-KGG). This modification can be detected using a commercially available commercially K-ε-GG antibody. Using this approach, approximately 1,000 SUMO2-modified Lys have been identified in HEK293T cells (75). The increasing interest in SUMOylation has promoted the development of novel techniques such as protease-reliant identification of SUMO modification (PRISM) (76). PRISM involves chemical blocking of all free Lys in a sample, followed by treatment with SUMO-specific proteases, and subsequent identification of the “freed” Lys by MS. This technique allows not only identification of SUMO sites on endogenous proteins but also changes in response to extracellular stimuli. Application of these novel techniques to the lymphocyte signaling field will surely generate new strategies to manipulate T cell function. Both ubiquitination and SUMOylation pose a bigger problem than phosphorylation because of their low relative abundance, making it harder to establish a threshold for noise vs biological relevance. Thus, tagged versions of these modifiers can increase the difference with the background. In this context, an interesting perspective will be the generation of mice bearing “knockin” tagged versions of these modifiers to identify substrates *in vivo*.

Protein *methylation* reduces the number of arginine hydrogen bond donors, weakening protein–protein and protein–nucleic acid interactions and thus, generating differential binding properties. While histone methylation plays a well-described role in chromatin remodeling and gene expression, other functional consequences of methylation are poorly defined. In this context, protein methylation could greatly benefit from global approaches to identify methylation sites that can be further subjected to hypothesis-driven experiments to determine the functional role of methylation in the regulation of protein function. To analyze Arg methylation in a global manner, a novel method consisting in isomethionine methyl-SILAC labeling coupled to antibody-mediated Arg-methylated peptide enrichment and subsequent identification by MS has been used to identify methylated peptides in human T cells. This approach identified 2,502 arginine methylation sites in TCR-stimulated Jurkat cells. Interestingly, chromatin remodeling machinery such as histone acetylases and deacetylases or Lys-methyltransferases were methylated in Arg residues, together with T cell antigen receptor signal machinery and several key transcription factors that regulate T cell fate determination (77). In addition, approaches to detect Lys methylation combining peptide immunoprecipitation with anti-methyl-Lys antibodies and MS detection have identified 552 lysine methylation sites on HeLa cells (78). Further work will be required to determine the role of specific methylations on T cell activation and differentiation, but these global approaches are opening the door to a new player on signal transduction pathways.

O-linked β-d-*N*-acetyl glucosamine (O-GlcNAc) addition (*O-GlcNAcylation*) is particular type of protein glycosylation on Ser/Thr residues, involved in diverse cellular metabolic and signaling pathways including T cell self-renewal and malignant transformation (79). As a tool to analyze O-GlcNAcylation in a global manner, a novel approach has been developed to isotopically label O-GlcNAc modifications. This method, based on feeding cells with ^13^C6-glucose, produces ^13^C-labeled UDP-GlcNAc from ^13^C6-glucose *via* the hexosamine biosynthetic pathway. In combination with O-GlcNAc peptide enrichment using phenylboronic acid solid phase extraction cartridge and MS identification, 105 O-GlcNAc and their dynamics have been determined on HeLa cells following this method (80). To summarize, the development of novel techniques for PTMs analysis in a global manner allows the identification of site-specific PTMs, and once the site is exposed, hypothesis-driven approaches can be applied to determine the role of that specific site on protein and cell functions.

## Conclusion

The overall conclusion of the work recapitulated here is that phosphoproteomic approaches have fulfilled their potential. They have been successfully applied in the identification of novel components of signaling networks, previously unknown kinase substrates and novel biological functions controlled by site-specific phosphorylation. These experimental approaches have also shed light into unexpected feedback mechanisms. In addition, already generated datasets will keep on generating new discoveries, since hundreds of site-specific phosphorylations on key molecules for T lymphocytes are waiting for further study of novel functions and regulatory mechanisms. From the initial work based on studies in cell lines, phosphoproteomics is now being successfully applied to more relevant experimental settings such as virus infection or analysis of tissue samples from T cell-mediated inflammatory diseases. Remarkably, this knowledge is unique to phosphoproteomics, since it will not have been generated using other experimental approaches.

## Perspectives

Phosphoproteomic analysis is ready to be taken into complicated experimental settings. An interesting yet unsolved question in the T lymphocyte signaling field is if effector T cells use unique components that contribute to the distinct outcome of TCR signaling in the different effector subtypes. The robustness of quantitative phosphoproteomic analysis can be used to examine TCR signaling in the different types of effector T lymphocytes in order to identify unique signaling events that can be used to manipulate the function of specific T cell subpopulations. In addition to novel components of signaling cascades, parameters such as stoichiometry of site-specific phosphorylations, abundance of signaling molecules, or even detailed kinetics on a particular phosphorylation event can be extracted from phosphoproteomics datasets. These parameters are often estimated from experimental systems, but phosphoproteomics datasets offer empirical data that can implement systems biology and mathematical modeling of signaling networks to make predictions and generate new hypothesis-driven experiments (81). These signaling features can shed light in conditions in which signaling is not qualitatively but quantitatively different: same players, different amounts. Quantitative differences of signaling components are the root to relevant biological phenomena such as the generation of heterogeneity (82) or signaling thresholds that control T cell responses (83, 84). As T cell function is strongly influenced by signals emanating from antigen receptor and cytokine/chemokine receptors, phosphoproteomics can be used to examine the crosstalk between different signals in a global manner, leading to the discovery of novel regulatory mechanisms and synergies. Moreover, simultaneous analysis of phosphorylations and other PTMs would surely generate a complete view of signaling cascades. The comprehensive knowledge of signaling cascades will generate better strategies for manipulation of T cell function in pathological conditions. Taking phosphoproteomics a step closer to the clinics, it can be used to identify drug action and resistance mechanism in kinase inhibitor treatments. Moreover, phosphoproteomic profiling of tumors can identify active kinases, knowledge that can be used for diagnosis implementation, treatment choice, and clinical development of kinase inhibitors (67).

## Author Contributions

All listed authors have made substantial contributions to the conception of the work, drafted the manuscript, and approved it for publication.

## Conflict of Interest Statement

The authors declare that the research was conducted in the absence of any commercial or financial relationships that could be construed as a potential conflict of interest.
